# Stent fractures in the superficial femoral artery: predisposing factors and their implications

**DOI:** 10.1590/1677-5449.202000142

**Published:** 2022-09-19

**Authors:** Fernando Trés Silveira, Álvaro Razuk, Paulo Fernandes Saad, Karen Ruggeri Saad, Gustavo José Politzer Telles, Pedro Ivo C. Ravizzini, Roberto Augusto Caffaro, Valter Castelli

**Affiliations:** 1 Faculdade de Ciências Médicas da Santa Casa de São Paulo – FCMSCSP, São Paulo, SP, Brasil.; 2 Universidade Federal de São Paulo – UNIFESP, São Paulo, SP, Brasil.; 3 Universidade Federal do Vale do São Francisco – UNIVASF, Petrolina, PE, Brasil.

**Keywords:** peripheral arterial disease, stents, endovascular procedures, doença arterial periférica, stents, procedimentos endovasculares

## Abstract

**Background:**

Fractures in stents implanted in the superficial femoral artery (SFA) are recognized complications of endovascular management of this arterial territory.

**Objectives:**

The objective of this study was to determine the prevalence of fractures in stents implanted in the SFA and to identify predisposing factors for these fractures together with their impact on the patency of these devices.

**Methods:**

The study included 39 patients (65.7±9.0 years) who previously underwent angioplasty for delivery of 56 stents into the SFA. During follow-up, which ranged from 7 to 46 months, variables were collected on the characteristics of the lesions treated and characteristics of the stents implanted. Two examiners independently analyzed digital radiographs for the presence of stent fractures and the patency of the devices.

**Results:**

We found a 10.7% prevalence of fracture of implanted stents. Implantation of multiple stents was identified as a significant predisposing factor for fractures. We observed a marked tendency for fractures in female patients and in lesions treated with longer stents (> 150 mm). Stenosis exceeding 50% and occlusions were significantly more frequent in fractured stents.

**Conclusions:**

This study suggests that implants longer than 150 mm and multiple stents are associated with higher device fracture rates. In cases with stent fractures, stenoses exceeding 50% and occlusions were significantly more frequent.

## Resumo

**Contexto:**

As fraturas de stents implantados na artéria femoral superficial (AFS) são uma complicação reconhecida pós-tratamento endovascular desse território arterial.

**Objetivos:**

Este estudo objetivou determinar a presença de fraturas nos stents implantados na AFS e identificar fatores predisponentes para essas fraturas, juntamente com o impacto na perviedade desses dispositivos.

**Métodos:**

Foram incluídos 39 pacientes (65,7±9,0 anos) previamente submetidos à angioplastia para colocação de 56 stents na AFS. Durante o seguimento, que variou de 7 a 46 meses, foram coletadas variáveis referentes às características das lesões tratadas e às características dos stents implantados. Dois examinadores analisaram radiografias digitais para verificar a presença de fraturas de stent e a perviedade dos dispositivos independentemente.

**Resultados:**

Foi encontrada uma prevalência de 10,7% de fratura nos stents implantados. O implante de múltiplos stents foi identificado como fator predisponente significativo para fraturas. Foi observada uma tendência acentuada de fraturas em pacientes do sexo feminino e em lesões tratadas com stents mais longos (> 150 mm). As estenoses acima de 50% e as oclusões foram significativamente mais frequentes em stents fraturados.

**Conclusões:**

Este estudo sugere que implantes de múltiplos stents ou de stents com extensão maior que 150 mm estão associados a maiores taxas de fraturas do dispositivo. No caso de fraturas de stents, as estenoses mais significativas que 50% e as oclusões foram consideravelmente mais frequentes.

## INTRODUCTION

The superficial femoral artery (SFA) and the popliteal artery are frequently targeted by atherosclerotic disease, leading to significant morbidity. The segments affected tend to present significant calcification and occlusion.^1^

Revascularization treatment of vascular lesions in peripheral arteries by stenting has demonstrated substantial advantages over conventional surgery in carefully selected cases.^2^ However, allied with these benefits are risks inherent to endovascular revascularization; the two most significant risks being restenosis and stent fracture. Restenosis constitutes the most typical unfavorable outcome, involving vessel narrowing at the site of surgery.^3^ Occurrence of fractures is variable and can impair stent patency, leading to restenosis, obstruction, formation of pseudoaneurysms, or embolization which cause complications and mortality over the short and long terms.^1^^,^^4^^-^6

Currently, there are other therapeutic options for femoropopliteal arterial disease. However, there is no clear consensus on the specific type of endovascular treatment most appropriate for each case. We are in a “leaving nothing behind” era. However, there are insufficient clinical data on patients with femoropopliteal disease treated using drug-coated balloons, because there is a lack of reliable prospective data over five years on this technology.^7^^,^^8^ Moreover, the latest FDA recommendations limiting use of pharmacological balloons suggest that use of stent technology should be revisited until the current recommendations are clarified.

A series of studies conducted since 2002 have raised concerns over the unexpectedly high prevalence of fractures of endovascular devices,^4^^,^^5^^,^^9^^-^11 despite the introduction of nitinol stents.^12^^,^^13^ Doubts still remain over long-term stent integrity and SFA patency, particularly concerning the real factors contributing to these stent fractures.^14^ The rationale for the present study was grounded in this line of clinical investigation, involving observation of the presence of fractures in stents placed in the SFA, analysis of potential predisposing factors for these fractures, and their impact on stent patency.

## METHODS

### Study design

This is an observational, prospective (follow-up) study of consecutive case studies. The research was approved by the Research Ethics Committee at our institution (decision number 5.525.582).

Between 2011 and 2012, 62 patients underwent superficial femoral artery angioplasty. The inclusion criterion was patients treated with angioplasty and placement of one or more stents in the superficial femoral artery. The exclusion criteria were failure to perform one or more of the data collection tests determined by the research protocol during follow-up. Thirty-nine patients were included in the study and none were excluded according to the criteria established.

### Data on the prior surgical procedure

All angioplasties with stent placement were performed by the surgeons of the vascular surgery team at this institution using the same technique (except for one case in which there was no pre-dilation before stent placement, and which made up the sample). We used nitinol stents to treat these patients, brands Maris (Medtronic, Minneapolis, US), Smart (Cordis, Santa Clara, US), EverFlex (EV3, Plymouth, US), and Vascuflex (B Braun, Melsungen, AL).

### Data collected

During follow-up consultations, a research team member filled out a form for each patient with the following variables: time elapsed between angioplasty and observation of the fracture; gender; age; presence of comorbidities or risk factors for PAD; site of lesions treated; classification according to Rutherford criteria; type of lesion according to TASC II consensus guidelines;^15^ and number and lengths of stents implanted.

After the consultation, patients were referred for digital radiography of the topography of the SFA to check the integrity of the stent (primary outcome) and examined with Doppler ultrasonography to assess stent patency (secondary outcome).

Digital X-rays of the SFA topography were produced in two radiographic planes. Fractures thus observed were classified according to the stent fracture types proposed by Allie et al.^12^ These authors classify fractures into four types: Type I: A single strut fracture only; Type II: multiple single nitinol stent fractures that can occur at different sites; Type III: multiple nitinol stent fractures resulting in a complete transverse linear fracture but without stent displacement and Type IV: a complete transverse linear type III fracture with stent displacement.

Doppler examinations were performed with an ultrasound device with a linear transducer by a single observer blinded to data on the presence or absence of stent fractures. We assessed stent patency according to peak systolic velocity (PSV). Stenosis greater than 50% was defined as when the stent exhibited a PSV of over 250 cm/s or a PSV 2.5 times greater than the velocity measured in the region proximal to the stent; velocities lower than this value were deemed non-significant stenosis. Stents with no flow indicated the presence of occlusion.

### Statistics

The data collected for this study were expressed as frequencies or as means and standard deviations. Fisher’s exact and chi-square tests were applied to analyze the variance of data expressed in frequencies. Student’s *t-*test adjusted for the variance of the subsamples (F-Test) was applied to analyze the variance of data expressed as means and standard deviations. A probability of 95% was adopted for rejection of the null hypothesis.

## RESULTS

No patients died during the study and there were no losses during follow-up of these patients. This clinical study sample comprised 17 (43.6%) men and 22 (56.4%) women with a mean age of 65.7±9.0 years. The mean ages of men (65.1±6.5 years) and women (66.7±10.6 years) proved homogeneous (p= 0.702).

Table 1 shows the patients' clinical presentations when indicated for angioplasty and stenting, their comorbidities, and the frequency of association between comorbidities and/or risk factors. The number of comorbidities or risk factors present in this patient group showed a weak positive correlation with patient age (r= 0.28).

**Table 1:** Distribution of frequencies related to comorbidities and clinical presentation of the 39 patients subjected to angioplasty with stenting of the superficial femoral artery.

**Presence of Comorbidities**		**n**	**%**
No comorbidities		7	17.9%
Multiple/single comorbidities	single comorbidity or risk factor	12	30.7%
	2 comorbidities and/or risk factors	14	35.9%
	3 comorbidities and/or risk factors	6	15.5%
	Systemic Arterial Hypertension	26	66.7%
	Diabetes Mellitus	20	51.3%
Comorbidities	Smoking	7	17.9%
	Dyslipidemia	5	12.8%
	Congestive heart failure	4	10.6%
**Clinical Presentation**		**n**	**%**
Critical limb ischemia		33	84.6%
Mild claudication or limiting claudication		6	15.4%
			

**Figure 1:**
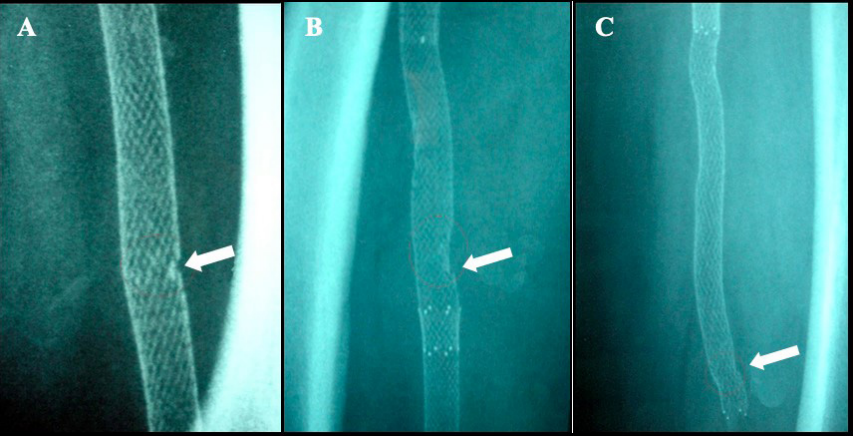
Radiology X-ray images of stent fractures (A) Type I. (B) Type II, and (C) Type III. Type I: A single strut fracture only; Type II: multiple single nitinol stent fractures that can occur at different sites; Type III: multiple nitinol stent fractures resulting in a complete transverse linear fracture but without stent displacement.^12^

Data describing the SFA lesions are given in Table 2. A total of 56 stents were implanted: 27 patients (69.2%) received a single stent; seven patients (17.9%) two stents; and five subjects (12.8%) were implanted with three stents. Six stent fractures were observed, representing 10.7% of the stents implanted in the SFA, or 15.3% of the patients treated. Three of the six fractures detected were classified as Type I, two as Type II, and one as Type III (Figure 1).

**Table 2:** Distribution of frequencies of characteristics of arterial lesions in 39 patients subjected to angioplasty with stenting of the superficial femoral artery.

**Characteristics of lesions**		**n**	**%**
Side affected	Right	18	46.2%
	Left	21	53.8%
Number of segments affected	Single segment	26	66.7%
	Two segments	8	20.5%
	Three segments	5	12.8%
Location of lesions	Proximal third	5	12.8%
	Middle third	4	10.3%
	Distal third	17	43.6%
	Proximal + middle thirds	1	2.6%
	Middle + distal thirds	7	17.9%
	Diffuse	5	12.8%
Rutherford Classification	1	1	2.6%
	3	5	12.8%
	4	3	7.7%
	5	26	66.7%
	6	4	10.2%
TASC	A	6	15.4%
	B	20	51.3%
	C	3	7.7%
	D	10	25.6%

The follow-up interval of these procedures, calculated between the date of angioplasty with stenting and the most recent Doppler ultrasonography, ranged from 7 to 46 months, with a mean of 23.5±10.4 months and a median of 21 months. No significant difference in mean follow-up time (p= 0.469) was found between patients with fractured stents (20.7±5.0 months) and patients with intact stents (24.1±11.1 months). No fractures were observed in the four patients followed up for up to 12 months, nor among the six patients followed for more than 37 months. Among the 20 patients followed up for between 13 and 24 months, 25% had fractures (accounting for 83.3% of stent fracture cases). Nine patients were followed up for between 25 and 36 months, one of whom (11.1%) had a stent fracture.

A trend was observed (Χ^2^ = 3.58; p= 0.058) for stent fractures to occur more frequently in female (22.7%) than in male patients (5.9%). The data show that 83.3% of the six fractures diagnosed were of stents implanted in women.

The mean age of patients diagnosed with stent fractures did not differ to that of patients without stent fractures (64.0±7.1 years and 66.0±9.3 years, respectively; F= 0.852; p= 0.616).

Frequencies distributed according to the presence or absence of stent fractures for the variables studied are shown in Table 3. Although 50% of the stent fractures had occurred in TASC II type D lesions, no association was found between occurrence of fractures and types of lesions according to these consensus guidelines (Χ^2^=3.91; p=0.271). No stent fractures were observed in the six cases of stenting for Type A lesions, while such fractures were seen in 10% of implantations performed for type B lesions, in 33.3% of type C lesion, and 30% of cases of type D lesions.

**Table 3:** Distribution of frequencies of comorbidities and/or risk factors in 39 patients subjected to angioplasty with stenting of the superficial femoral artery, by presence or absence of fracture of stents implanted.

**Variables**	**Stent fracture**				**Totals** **(n = 39)**		**p-value**
	**Present** **(n = 6)**		**Absent** **(n = 33)**				
	**n**	**%**	**n**	**%**	**N**	**%**	
Isolated or associated comorbidities and/or risk factors							
Systemic arterial hypertension	4	66.7%	22	66.7%	26	66.7%	
Diabetes mellitus	2	33.3%	18	54.5%	20	51.5%	
Congestive heart failure	-	-	1	3.0%	1	2.6%	
Smoking	-	-	7	21.2%	7	17.9%	
Dyslipidemia	1	16.7%	4	12.1%	5	12.8%	0.385
Side of lesion							
Right	2	33.3%	16	48.5%	18	46.1%	
Left	4	66.7%	17	51.5%	21	53.9%	0.257
Location of lesions							
Proximal third	-	-	5	15.1%	5	12.9%	
Middle third	-	-	4	12.2%	4	10.2%	
Distal third	3	50.0%	14	42.4%	17	43.5%	
Proximal/middle thirds	1	16.7%	-	-	1	2.6%	
Middle/distal thirds	1	16.7%	6	18.1%	7	17.9%	
Diffuse	1	16.7%	4	12.2%	5	12.9%	0.324
Rutherford Classification							
1-4	-	-	9	27.3%	9	23.1%	
5-6	6	100.0%	24	72.7%	30	76.9%	0.047^*^
TASC II consensus guidelines							
A	-	-	6	18.2%	6	15.4%	
B	2	33.3%	18	54.5%	20	51.3%	
C	1	16.7%	2	6.1%	3	7.7%	
D	3	50.0%	7	21.2%	10	25.6%	0.271
Number of stents implanted							
1	2	33.3%	25	75.7%	27	69.2%	
2-3	4	66.7%	8	24.3%	12	30.8%	0.010*
Length							
< 150 mm	2	33.3%	24	72.7%	26	66.7%	
> 150 mm	4	66.7%	9	27.3%	13	33.3	0.076
Sonographic findings							
Stenosis < 50%	-	-	19	57.6%	1	48.7%	
Stenosis > 50%	2	33.3%	6	18.2%	8	20.5%	
Occlusion	4	66.7%	8	24.2%	12	30.7%	0.030*

* Statistically significant values.

A significantly lower frequency (Χ^2^=6.51; p=0.010) of fractures was detected in lesions treated using a single stent. For the overall sample, the mean length of the implanted stent was 163.8±81.7mm (40 to 370mm), and the median length was 150mm. A trend (p=0.066) was observed for stents with fractures to have longer mean lengths (220.0±75.4mm) compared to stents without fractures (153.6±79.6mm). The distribution of fractured stents versus intact stents by shorter or longer lengths confirmed this trend (Χ^2^ = 3.14; p = 0.076). Concerning these findings, it is noteworthy that no stent fractures were found among the 16 patients with implantations < 150mm in length, whereas 26% of the 23 patients with implantations > 150mm in length had stent fractures. Nevertheless, the results show that fractures in stents implanted in the SFA are more strongly associated with the number of stents implanted than with the total length of these implantations.

## DISCUSSION

The cumulative incidence of fractured stents implanted in the femoropopliteal segment varies from 2 to 65% of stents implanted, with higher rates reported in studies that explicitly seek to determine the frequency of this occurrence.^1^^,^^4^^-^6^,^^11^^,^^13^^,^^16^^-^19 These fractures have been associated with several different anatomic and clinical variables and with characteristics of the stents deployed.

The prevalence of fracture occurrence in the present study was 15.3% of all patients treated and 10.7% of all stents implanted. Most studies report fracture rates of between 14 and 27%,^4^^,^^5^^,^^16^^-^19 where these rates may be higher or lower depending on the device brand or material. However, no significant differences in occurrence of these fractures have been reported in comparisons of different brand devices.^16^^,^^19^ A lower prevalence of fractures was observed in our sample (10.7%), based on the proportion of all stents implanted, which is the measure adopted by most studies to calculate this rate. Only Schillinger et al.^20^ reported a lower rate, of fractures in 2% of nitinol stents implanted in SFAs.

We failed to establish any relationships between fracture and stent type or patency. This was most likely due to the relatively small number of fractures and the fact that all the fractured stents were either occluded or had stenosis exceeding 50%. On the other hand, Iida et al.^19^ state that there are differences in the outcomes of SFA cases depending on the fracture type, having found type 2 fractures to exert a potentially more significant adverse effect on the patency of these devices, while considering type 1 and type 3 fractures to be benign. In contrast, Scheinert et al.^21^ reported a strong correlation between obstruction and fracture type, finding type 3 fractures to be significantly more deleterious to device patency than type 2.

We observed that the critical period for occurrence of device fractures is the second year after endovascular treatment, since 83.3% of fractures were identified in our patients at 13 to 24 weeks’ follow-up. Duda et al.^5^^,^^9^ reported fracture incidences of 17% and 26% after six and 18 months’ follow-up, respectively. However, Scheinert et al.^21^ called attention to the fact that stent fractures occurred at different time intervals after implantation depending on type and brand of device used. These factors have been the focus of *in vitro* analyses of deformation,^22^^,^^23^ fatigue, and durability of stents.^24^ This calls for standardized clinical studies to elucidate the issue of the estimated critical period for occurrence of fractures in different types of devices used for revascularization of the SFA.

PAD is known to be more frequent in male patients, although the disparity compared to female patients tends to reduce with advancing age, particularly after 70 years of age.^25^ Indeed, in most studies on fractures and/or patency of stents implanted in the SFA, around 70% of each sample comprised male subjects.^13^^,^^16^^-^19^,^^26^ However, none of these studies reported the frequency of stent fractures by patient gender, except for the study by Iida et al.,^13^ in which stent fractures were observed in one out of eight women (13%) and in 10 out of 32 men (31%), in a sample containing 80% male patients. In contrast to the studies cited, the present study sample contained an almost equal proportion of men (44%) and women (56%), with a slightly higher frequency of females. A tendency was observed for a more frequent occurrence of stent fractures among female patients. Since this observation was not reported by other studies that served as the basis of our research, this result may initially be attributed to casual findings. It is also necessary to consider that the small sample size is a limitation of this study.

However, this variable warrants further investigation in future studies since, although PAD is more frequent in men, the female gender may ultimately be confirmed or refuted as a predisposing factor for fractures in stents implanted in the SFA.

The mean age, number and type of comorbidities, and risk factors of the patients assessed were similar among those with and without stent fractures. These findings corroborate the observations of other researchers reporting fractures in devices implanted in the SFA.^13^^,^^16^^,^^17^^,^^19^^,^^20^^,^^26^ Moreover, according to a systematic review of contributing factors, these variables exerted no significant influence on the occurrence of fractures in stents implanted for coronary revascularization.^6^

Similarly, no association was found between lesion side and site of the treated limb with the occurrence of fractures in implanted devices. There is no evidence in the literature consistent with such an influence. Nevertheless, there are some reports of significantly more frequent involvement of the distal third of the SFA in patients with stent fracture.^13^ In this study, we detected that 50% of fractures were in stents used to treat injuries in this location, which was also the most common injury site, representing 43.5% of cases.

Considering all the Rutherford classification categories, no differences were found between patients with fractured stents and those with intact stents. On the other hand, stratifying patients into two groups corresponding to lower (1 to 4) and higher (5 and 6) categories on the Rutherford classification revealed a significantly higher frequency of device fracture in the higher category group, suggesting a predisposing role in fractures. These findings conflict with results reported by Iida et al.^17^ Fracture of devices implanted in the femoropopliteal segment was significantly associated with lower average Rutherford classification categories. These authors' analysis was based on the mean Rutherford classifications for fractured stents (3.0±0.7) and intact stents (3.3±1.2). In the same study, however, the authors found a significantly higher frequency of fractures in patients with less severe ischemia of the lower limbs but did not discuss these findings.

In the present study, TASC II lesion types were not associated with stent fractures, although 50% of fractures were detected in type D lesions. The results of previous studies are conflicting on this point. In an initial study, Iida et al.^17^ found a significantly higher prevalence of implant fractures among patients with type D lesions, but in a subsequent study they failed to observe any differences between lesion types.

Duda et al.^4^^,^^5^^,^^9^ observed a strong association between the number of devices implanted and the occurrence of fractures, recommending delivery of two devices at most. Likewise, Iida et al.^13^^,^^17^^,^^19^ confirmed that the greater the number of stents implanted, the higher the frequency of fractures. Our findings confirm that the number of stents implanted is a relevant risk factor for occurrence of fractures.

The length of the revascularized segment has been considered an unequivocal risk factor for fatigue and resultant fracture of stents in femoral circulation. Scheinert et al.^21^ reported a 13.2% risk of fracture in stents < 8cm in length, 42.4% in longer stents, and 52.0% for stents measuring more than 16cm. The SIROCCO II trial results reported fractures in 18% of devices with an average length of 81.5mm over a six-month follow-up.^5^ Conversely, in a 2006 study, Iida et al.^13^ reported no association between length of the revascularized segment and fractures in devices implanted in the SFA. However, in a 2011 study by the same authors, the mean lengths of stents implanted in the femoropopliteal segment were 208mm and 121mm for fractured versus intact stents, respectively.^19^ In our sample, there was a trend for revascularized segment length to be associated with fracture; none of the implants with lengths < 150mm exhibited fractures, whereas fractures were detected in 26% of implants with lengths > 150mm.

Restenosis and occlusion secondary to fractured stents result in recurrence of symptoms and the need for further revascularization intervention. Scheinert et al.^21^ found restenosis and obstruction in 32.8% and 34.4% of fractured devices, respectively, in a sample of 261 revascularizations with 27.5% fractured stents. Chronic total occlusion was found in 82% of fractured stents versus 31% of intact stents in a sample of 40 patients.^13^ All cases of device fracture in our study progressed either to stenosis > 50% (33.3%) or obstruction (66.7%), associating these outcomes with stent fracture.

This clinical study's findings contribute to knowledge on the occurrence of fractures in stents implanted to the SFA, revealing a 10.7% prevalence of fractured stents, predominantly in cases of multiple stent implants for the treatment of arterial disease (Rutherford 5 and 6). These fractures invariably led to > 50% stenosis or occlusion of the fractured device. These results also indicate a need for further investigations to confirm the role of female gender and device length as predisposing factors for stent fracture.

The limitation of this study is related to the small sample size. The sample calculation for this study suggested a sample comprising 68 patients. We calculated the sample number based on a projection of the symptomatic Brazilian population as 6,000,000 people, a 10% sampling error, and a 90% confidence level.

## CONCLUSIONS

This study suggests that implants longer than 150 mm or multiple stents are associated with higher device fracture rates. Stenoses exceeding 50%, and occlusions were significantly more frequent in cases with stent fractures.
